# Amputees’ Attitudes Toward Participation in Amputee Support Groups and the Role of Virtual Technology in Supporting Amputees: Survey Study

**DOI:** 10.2196/14887

**Published:** 2019-08-29

**Authors:** Edward P Nathan, Sandra L Winkler

**Affiliations:** 1 Roy H Park School of Communications Ithaca College Ithaca, NY United States; 2 Research Service James A Haley Veterans Affairs Hospital Tampa, FL United States; 3 Occupational Therapy Department Nova Southeastern University Fort Lauderdale, FL United States

**Keywords:** amputation, amputee, survey, support, support group, technology, virtual, virtual reality

## Abstract

**Background:**

Acquiring information about and living with an amputation (or limb differential) is a lifelong endeavor. Although medical institutions address the immediate medical needs of amputees, information regarding how to live life as an amputee is provided from numerous sources, one of which is amputee support groups.

**Objective:**

This study aimed at understanding why amputees join support groups, leave support groups, and possibly return to support groups as well as how technology, specifically virtual reality, might play a role in supporting patients’ needs. The results are intended to provide data for support groups, to increase their impact on amputee participants.

**Methods:**

A 38-item online survey was developed based on the findings of a previous randomized trial. The survey was administered between April and September 2018 and divided into four sections: Demographics, Limb Loss History, Amputee Support Group Participation, and Technology Usage. Items used multiple-choice, drop-down menu, check-box formats with explanation boxes for open-ended responses. Descriptive analyses were performed for both qualitative (open-ended questions) and quantitative data.

**Results:**

Of the 59 amputees enrolled, 54 completed the survey. All the respondents were aged 20-39 years, and nearly half of the older respondents thought audio and video teleconferencing or avatar-based technology would increase participation in support groups. The results suggest that an early goal for amputees who join support groups is to focus on regaining mobility and functionality in order to return to their normal life. Once achieved, the goal transitions to one of social connection with other amputees, although there is a caveat: Simply being an amputee may not provide sufficient connections for developing long-term social relationships. The strongest reason for joining a support group was to learn about living with an amputation, followed by networking and learning new skills.

**Conclusions:**

The results suggest four key takeaways regarding amputee participation in support groups: (1) the needs of participants in amputee support groups change over time; (2) meeting content needs to be relevant to agendas primarily driven by participants; (3) support group participation is also driven by the desire to increase functionality by developing skills, become familiar with prosthetic technology, have more than amputation in common with other participants, and participate at the designated meeting time and location; and (4) the use of technology should support patients’ needs.

## Introduction

Acquiring current and evolving prosthetic and health-related information is an ongoing process throughout the lifespan of an amputee. Although the availability of global data regarding the incidence of amputation is varied and nonstandardized, it is estimated that there are nearly 2 million people living with limb loss in the United States, [1] with approximately 185,000 amputations occurring here each year [2]. Worldwide, peer support is a viable venue for acquiring and sharing this information. A support group is defined as a group of people with common experiences and concerns who provide emotional and moral support for one another [3]. The concept of patient support groups dates back to the late 18th century France, where “The governor of Bicêtre Hospital in Paris, Jean Baptiste Pussin, recognized the value of employing recovered patients as hospital staff. The chief physician at the hospital, Philippe Pinel praised these peer staff for being ‘gentle, honest, and humane’, ‘averse from active cruelty’, and ‘disposed to kindness’” [4].

The power and impact of support groups were demonstrated by one of the earliest support groups, Alcoholics Anonymous, in 1935. Alcoholics Anonymous showed how self-help groups could do what the medical profession had, for the most part, been unable to do, which was to help alcoholics successfully manage their addiction [5]. In the latter half of the 20th century, support groups in both the mental health field and medical profession proliferated. Support groups were created to help those affected by numerous conditions, from addictions to heart disease, cancer, and grief support. One such group—the Amputee Coalition—was founded in 1986 when “a small group of amputee support group leaders recognized the need for an organization dedicated to the needs of people with limb loss, their families and healthcare providers” [6]. Peer support for amputees can assist with adjustment to amputation; psychosocial healing [7]; and sharing information about medical support, adaptive tools, and mental health resources.

Although traditional peer-to-peer support groups have functioned in face-to-face, real-time meetings, a limitation of face-to-face peer support groups is the lack of access due to distance, time, transportation, etc. This is especially true for individuals with disabilities, chronic illness, or mental illness [8]. These populations may not have the physical or social resources to participate in face-to-face support groups. As a result, virtual health care support groups are a potential alternative. Virtual health care support groups utilize the communication technology of virtual worlds. The growth and positive impact of virtual worlds has created many new possibilities for amputee support groups. A 2013 study of 196 individuals with physical or mental disabilities who actively participated in Virtual Ability in the Second Life virtual world found an increase in self-esteem, social support, and life satisfaction [9].

Characteristics of virtual worlds include persistence, anonymity, 24/7 access to individuals globally, and virtual embodiment [8]. Persistence is the ability of the virtual environment to continue to operate, use, and collect data irrespective of whether individuals are interacting with it via their avatars [8]. Virtual worlds are anonymous because the use of avatars allows the user to mask their identity, which includes the ability to alter their age, gender, physical appearance, and other characteristics including disabilities. Virtual worlds allow amputees to interact globally, overcoming geographic limitations and isolation. Virtual embodiment allows users to interact with their virtual geography including other individuals and objects in the environment and in the virtual world [10]. In other words, the virtual world environment may allow people to participate in support group sessions with a level of access and anonymity that is not possible in a face-to-face support group setting.

**Figure 1:**
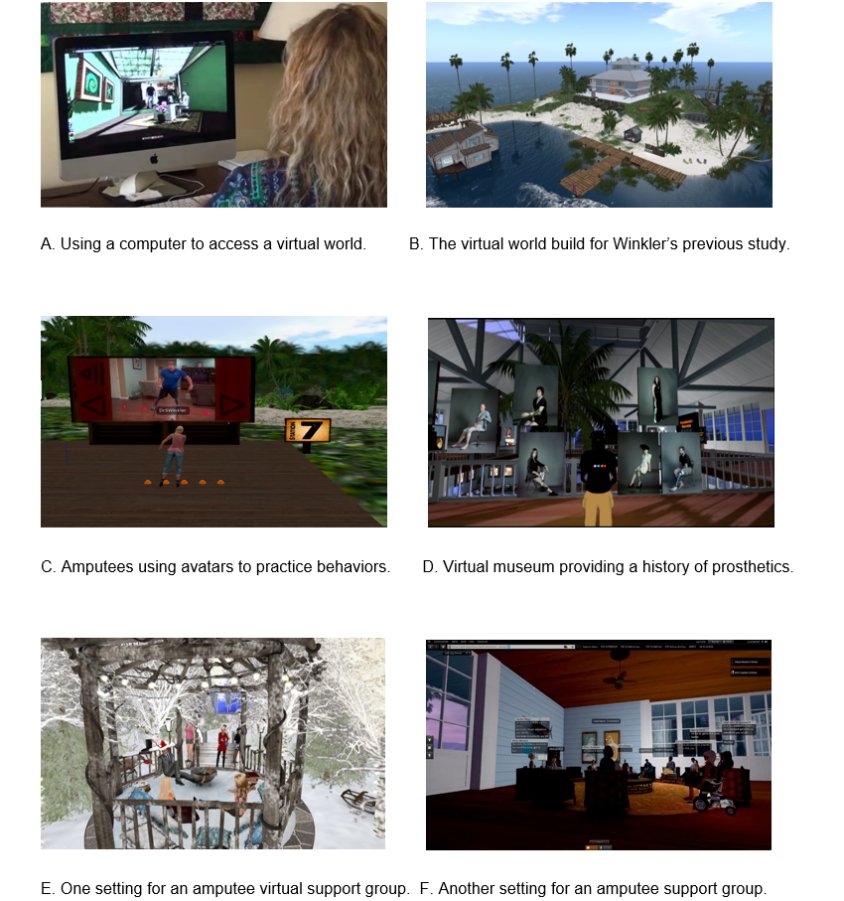
(A) User engaging with a virtual world via computer, (B) the virtual island, (C) practicing balance activities, (D) a virtual museum, and participating in regularly scheduled (E), and holiday virtual support groups (F).

Winkler et al [11] tested the use of a virtual environment to provide self-management information including skill development to amputees. Figure 1A shows how a computer is used to access the virtual world via the internet. Figure 1B shows the virtual world built for Winkler’s previous study [11]. Amputees had the opportunity to view themselves as an avatar, practicing desired behaviors such as balance (Figure 1C) and providing information on the history of prosthetics (Figure 1D). Figure 1E and F show virtual support groups. Some participants performed activities wearing a prosthetic limb and socialized with other amputees virtually, before having a prosthetic limb and interacting with other amputees in real life. Attempts at convening a virtual support group within the virtual world infrastructure developed by Winkler et al [11] were not sustained, which was the impetus for the survey study reported in this paper. Thus, the purpose of the survey study was to understand what amputees seek in a support group and to measure the acceptability of using technology to increase access to support groups. More specifically, the study sought to answer four research questions:

1. Why do amputees join support groups?
2. Why do amputees not participate in support groups?
3. Why do amputees rejoin support groups?
4. Is there a role for virtual technology in improving amputee support group engagement?

## Methods

This study was approved by the Nova Southeastern University Institutional Review Board. A cross-sectional survey design was used to survey a sample of amputees with an email address, as the survey was administered by email and required internet access. Using convenience sampling, amputees were recruited using an institutional review board­–approved flyer distributed on the Amputee Coalition website and Facebook page; InMotion magazine; and at the 2018 Amputee Coalition annual conference in Tucson, AZ. Interested participants were instructed to contact the first author by phone or email. Study data were collected and managed using Research Electronic Data Capture (REDCap) electronic data capturing tools hosted at Nova Southeastern University. REDCap is a secure, Web-based app designed to support data capture for research studies, providing an intuitive interface for validating data entry; audit trails for tracking data manipulation and export procedures; automated export procedures for seamless data downloads to common statistical packages; and procedures for importing data from external sources [12]. Subjects who decided to participate in the study provided their name and email to the first author. They were then sent an email via REDCap with a link to the closed survey. A statement of consent preceded the survey. Respondents were able to review and change their answers.

The 38-item survey administered between April and September 2018 was divided into four section headers: Demographics, Limb Loss History, Amputee Support Group Participation, and Technology Usage. The Demographic section of the survey had four demographic questions including gender, race-ethnicity, age, and military service. The Limb Loss History section had seven questions including type, cause, and number of participant amputations. The Amputee Support Group Participation section included questions about the number, type, and frequency of support group participation. The Technology Usage portion of the survey asked participants about the type, frequency, and access to various types of technology. The formats of the items were multiple-choice, drop-down menu, check boxes (designated for single and multiple answers). Explanation boxes were also provided for some questions to give participants a chance to choose the “other” option and provide open-ended responses.

There were additional comments sections for respondents to provide further, optional information. Qualitative descriptive analyses were used for open-ended questions. Quantitative statistical analysis was applied to the numerical data captured from the survey.

## Results

Fifty-nine amputees were enrolled in the study. Data were analyzed for the 54 amputees who completed the survey, although most, not all, items were completed for all participants. Tables 1 and 2 present the demographic composition of the participant group.

The first research question asked why amputees join support groups. Table 3 shows how many participants belong to support groups and other information about support group participation.

**Table 1:** Demographic data.

Variable		Sample (N=54)
Age (years), mean (range)		58.6 (20-82)^a^
**Sex, n (%)**		
	Male	35 (65)^b^
	Male LGBT^b^	1 (2)
	Female	18 (33)
**Race, n (%)**		
	Caucasian/White	50 (93)
	Black/African American	3 (6)
	Native American	1 (2)
**Ethnicity, n (%)**		
	Latino/Latina	2 (4)
	Not Latino/Latina	52 (96)
**Military service, n (%)**		
	Yes	8 (15)
	No	46 (85)
**Number of amputations, n (%)**		
	1	41 (76)
	2	8 (15)
	3	2 (4)
	>4	3 (6)

^a^Continuous variable in age (range).

^b^LGBT: lesbian, gay, bisexual, transgender.

**Table 2:** Amputation data.

Variable		Value, n (%)
**Number of amputations (54 subjects)**		
	1	41 (76)
	2	8 (15)
	3	2 (4)
	≥4	3 (6)
**Amputation type (total of 64 amputations, as some subjects had more than one amputation)**		
	Below knee	35 (55)
	Above knee	11 (17)
	Finger(s)	6 (9)
	Below elbow	3 (5)
	Shoulder disarticulation	1 (2)
	Above elbow	1 (2)
	Elbow disarticulation	1 (2)
	Hip disarticulation	1 (2)
	Knee disarticulation	1 (2)
	Foot	1 (2)
	Toe(s)	1 (2)
**Amputation side**		
	Left	28 (52)
	Right	20 (37)
	Bilateral	4 (7)
	Quadrimembral	2 (4)
**Amputation cause (total of 64 amputations, some subjects had more than one cause)**		
	Trauma	18 (28)
	Infection	14 (22)
	Diabetes related	10 (16)
	Other	8 (13)
	Vascular disease	4 (6)
	Cancer	4 (6)
	Disease related	3 (5)
	Congenital/birth	2 (3)
	Unknown	1 (2)
**Time since most recent amputation (years; N=53, as one participant did not respond**		
	<1	4 (8)
	1-2	9 (17)
	2-3	8 (15)
	3-5	8 (15)
	5-10	11 (21)
	≥11	13 (25)

**Table 3:** Amputee support group experience.

Experience		Participants, n (%)
**Belong to at least one support group**		
	Yes	45 (83)
	No	9 (17)
**Number of years belonged to support group (45 responses)**		
	<1	11 (24)
	1-2	9 (20)
	2-3	8 (18)
	3-5	9 (20)
	5-10	6 (13)
	≥11	2 (4)
**Number of support groups participated in the past 12 months (one missing value, N=53 respondents)**		
	0	14 (26)
	1	23 (43)
	2	10 (19)
	3	4 (8)
	>3	2 (4)
**Frequency of support group meets (47 responses)**		
	Once a month	30 (64)
	Once every 2 months	4 (9)
	Once every 3 months	2 (4)
	Twice a year	1 (2)
	Other	10 (21)
**Number of meetings attended past 12 months (primary group; 47 responses)**		
	0	9 (19)
	1-3	17 (36)
	4-6	5 (11)
	7-9	5 (11)
	10-12	11 (23)

Questions 22, 24, and 26 in the 38-item participant survey asked respondents to select all applicable choices; therefore there could be multiple responses per participant.

Table 4 shows seven defined reasons (plus “Other”) for amputees’ participation in support groups, stratified by sex and military experience. The top reasons for all participants to join their support group were to obtain information about living with an amputation and to network with other amputees and health care professionals. For the group of military respondents, the top reasons were to obtain information about living with an amputation, to learn new skills as an amputee, and to network with other amputees and health care professionals.

Table 5 presents 13 reasons why amputees do not participate in support groups, stratified by sex and military experience. The top two reasons for all participants not to participate in support groups were because they did not know there was a support group close to them or the support group was not close enough. For the group of military respondents, the top reasons they did not participate was that they did not know there was a support group close to them, the support group was not close enough, or they did not feel they had anything in common with other members of the group. The “Other” option was the most frequent selection. Textbox 1 groups the “Other” reasons by theme.

**Table 4:** Reasons why participants decided to participate in their amputee support group (total responses=160).

Reason	All responses, n (%)	Male responses, n (%)	Female responses, n (%)	Military responses, n (%)
Obtain info about living with an amputation	34 (21.3)	23 (20.4)	11 (23.4)	7 (29.2)
Network with other amputees & health care professionals	30 (18.8)	20 (17.7)	10 (21.3)	4 (16.7)
Make new friends	21 (13.1)	14 (12.4)	7 (14.9)	2 (8.3)
Learn about new prosthetic technologies	19 (11.9)	16 (14.2)	3 (6.4)	1 (4.2)
Learn new skills as an amputee	19 (11.9)	12 (10.6)	7 (14.9)	5 (20.8)
Learn about new amputee support services	16 (10.0)	12 (10.6)	4 (8.5)	1 (4.2)
Learn about new amputee support technologies	13 (8.1)	10 (8.8)	3 (6.4)	2 (8.3)
Other	8 (5.0)	6 (5.3)	2 (4.3)	2 (8.3)

**Table 5:** Reasons for not participating in an amputee support group (total responses=69).

Reason	All responses, n (%)	Male responses, n (%)	Female responses, n (%)	Military responses, n (%)
I don’t know if there are any support groups near me	9 (13.0)	7 (16.7)	2 (7.4)	4 (23.5)
No amputee support groups are reasonably close to where I live	8 (11.6)	3 (7.1)	5 (18.5)	2 (11.8)
I don’t feel I have a lot in common with the other participants	7 (10.1)	6 (14.3)	1 (3.7)	2 (11.8)
The amputee support group meeting time does not work for me	7 (10.1)	5 (11.9)	2 (7.4)	0 (0.0)
Usually, not enough people show up	5 (7.2)	3 (7.1)	2 (7.4)	1 (5.9)
It’s always the same people who attend	5 (7.2)	3 (7.1)	2 (7.4)	1 (5.9)
The meeting topics are usually not relevant to my needs	5 (7.2)	3 (7.1)	2 (7.4)	1 (5.9)
Most of the participants are not in my age group	5 (7.2)	3 (7.1)	2 (7.4)	0 (0.0)
The meeting topics are usually not interesting to me	3 (4.3)	1 (2.4)	2 (7.4)	1 (5.9)
I do not like the amputee support group leadership	3 (4.3)	1 (2.4)	2 (7.4)	1 (5.9)
The healthcare professionals take up most of the meeting time	2 (2.9)	2 (4.8)	0 (0.0)	1 (5.9)
There is/are amputee support group(s) close to me, but I have no way to get there	0 (0.0)	0 (0.0)	0 (0.0)	0 (0.0)
Usually there are too many people	0 (0.0)	0 (0.0)	0 (0.0)	0 (0.0)
Other	10 (14.5)	5 (11.9)	5 (18.5)	3 (17.6)

**Other reasons for not participating in support groups.**

**Time:**

- Meetings are held every other month and I lose track of which month they are held on also I have many doctors’ appointments at the same time they are held on too.
- Wasn't able to make last one
- Limited time
- The support group for upper extremity amputees is only held twice a year. Unfortunately, I have missed meetings because the days did not work because of other obligations, or timing

**Gender:**

- I am the only woman except sometimes the wives of some amputees
- Being an above the elbow amputee and a women, I have found that most support groups are made up of primarily leg amputees and men

**Need:**

- Don't need support, can make this adjustment on my own, too proud, shows signs of weakness, won't help
- We had so many below 40 amputees we helped and convinced a few to start a “young” amputees support group and had joint meetings occasionally

- Never really thought about joining one.

**Fear:**

- I'm still embarrassed of my situation.

**Distance:**

- The support group for upper extremity amputees is 2 hours from my home

**Leadership and group process:**

- There is little, if any, opportunity provided for interchange of experience among the amputees. All of us, occasionally, wonder about this or that and if others have had similar experiences. The group does not even go around the table each time to introduce oneself and, perhaps, indicate the reasons for their amputation. In short, we know almost nothing about each other. The professionals make presentations and don't even ask if there are questions or how the presentation might be relevant to anyone in the room.

**Commonality:**

- I found my local support group to be a lot of older amputee people who were very negative and who is me type people who complained a lot instead of going out and doing things. I lead a much more active lifestyle than they do.

Table 6 shows 10 possible reasons why participants would return to a support group. The top reasons were that members of the group should have more in common with the respondent, the group should be closer geographically, and the topics should be more relevant. For the group of military respondents, the top reasons were that the group should have more in common with the respondent and the topics should be more relevant. Reasons specific to men were a preference that health care professionals not dominate support group meetings.

An ad hoc analysis looked at the relationships between the duration of participation in support groups and time since amputation for a cohort of 34 amputees who had a single amputation and belonged to a support group (Table 7). The data show that while about half the amputees dropped out of support groups as time since amputation increased, others joined support groups ≥5 years after their amputation. 

Table 8 shows the confidence level of using technology by age group. The majority of respondents rated themselves as “Very Confident” in using technology and used technology daily. Nearly 100% of all age groups used technology daily (not graphed). Only one respondent used technology “Weekly” (≥60 years) and one responded, “Not at all” (40-59 years).

All the participants in the 20-39 years age group and less than half of the older groups reported that they thought technology (teleconferencing, videoconferencing, and virtual environments) could increase participation in amputee support groups (Table 9).

Tables 10-12 compare the likeliness of joining a support group using teleconferencing, videoconferencing, and avatars by age group: 20% of the respondents aged 20-39 years were very likely to participate in support groups that use teleconferencing and videoconferencing. In comparison, 40% of those aged 20-39 years were very likely to participate in support groups that used avatars. Only 20% of those aged 40-59 years and ≥60 years responded that they were not at all likely to use teleconferencing or videoconferencing; 30% said they were not at all likely to use avatars.

**Table 6:** Reasons to participate in a support group (total responses=52).

Reason	All responses, n (%)	Male responses, n (%)	Female responses, n (%)	Military responses, n (%)
Participants that have more in common with me	13 (25.0)	7 (23.3)	6 (27.3)	3 (18.8)
An amputee support group closer to where I live	7 (13.5)	4 (13.3)	3 (13.6)	1 (6.3)
More relevant topics to my needs	7 (13.5)	3 (10.0)	4 (18.2)	3 (18.8)
Participants who were closer to my own age	6 (11.5)	3 (10.0)	3 (13.6)	1 (6.3)
Healthcare professionals who support the meetings without dominating the meetings	5 (9.6)	5 (16.7)	0 (0)	2 (12.5)
Other	5 (9.6)	3 (10.0)	2 (9.1)	1 (6.3)
The availability of technology like teleconferencing , videoconferencing, etc to be able to meet virtually	4 (7.7)	2 (6.7)	2 (9.1)	2 (12.5)
A larger group of participants	4 (7.7)	2 (6.7)	2 (9.1)	2 (12.5)
A smaller group of participants	1 (1.9)	1 (3.3)	0 (0)	1 (6.3)
If I could access low cost reliable transportation to get me to the meeting	0 (0)	0 (0)	0 (0)	0 (0)

**Table 7:** Relationship between time since amputation and duration of support group participation (total=34 hours).

Number of years since amputation	Number of years in support					
	<1	1-2	2-3	3-5	5-10	>11
<1	2	—^a^	—	—	—	—
1-2	3	3	—	—	—	—
2-3	0	2	4	—	—	—
3-5	3	0	2	3	—	—
5-10	0	1	0	3	1	—
>11	0	2	1	0	3	1

^a^Not applicable.

**Table 8:** Confidence in using technology (N=54).

Level of confidence	All participants, n (%)	Group: 20-39 years, n (%)	Group: 40-59 years, n (%)	Group: 60-82 years, n (%)
Very confident	25 (46.3)	3 (60.0)	12 (57.1)	10 (35.7)
Confident	17 (31.5)	1 (20.0)	7 (33.3)	9 (32.1)
Slightly confident	11 (20.4)	1 (20.0)	1 (4.8)	9 (32.1)
Not confident at all	1 (1.9)	0 (0.0)	1 (4.8)	0 (0.0)

**Table 9:** Responses to the question, “Can technology increase participation in amputee support groups?” (N=51).

Response	All participants, n (%)	Group: 20-39 years, n (%)	Group: 40-59 years^a^, n (%)	Group: 60-82 years^b^, n (%)
I think technology will increase the level of participation in amputee support groups.	23 (45.1)	5 (100)	9 (45.0)	9 (34.6)
I am not sure whether technology will increase the level of participation in amputee support groups.	17 (33.3)	0 (0)	7 (35.0)	10 (38.5)
I don't think technology will increase the level of participation in amputee support groups.	11 (21.6)	0 (0)	4 (20.0)	7 (26.9)

^a^One person did not respond.

^b^Two people did not respond.

**Table 10:** Likelihood of using teleconferencing to participate in an amputee support group (N=54).

Likelihood	All participants, n (%)	Group: 20-39 years, n (%)	Group: 40-59 years, n (%)	Group: 60-82 years, n (%)
Very likely	9 (16.7)	1 (20.0)	3 (14.3)	5 (17.9)
Somewhat likely	16 (29.6)	2 (40.0)	6 (28.6)	8 (28.6)
Neutral	13 (24.1)	2 (40.0)	5 (23.8)	6 (21.4)
Somewhat unlikely	6 (11.1)	0 (0)	3 (14.3)	3 (10.7)
Not at all likely	9 (16.7)	0 (0)	4 (19.0)	5 (17.9)
Don’t know what teleconferencing is	1 (1.9)	0 (0)	0 (0.0)	1 (3.6)

**Table 11:** Likelihood of using videoconferencing to participate in an amputee support group (N=54).

Likelihood	All participants, n (%)	Group: 20-39 years, n (%)	Group: 40-59 years, n (%)	Group: 60-82 years, n (%)
Very likely	7 (13.0)	1 (20.0)	3 (14.3)	3 (10.7)
Somewhat likely	15 (27.8)	1 (20.0)	4 (19.0)	10 (35.7)
Neutral	16 (29.6)	3 (60.0)	7 (33.3)	6 (21.4)
Somewhat unlikely	5 (9.3)	0 (0.0)	2 (9.5)	3 (10.7)
Not at all likely	10 (18.5)	0 (0.0)	5 (23.8)	5 (17.9)
I don’t know what teleconferencing is	1 (1.9)	0 (0.0)	0 (0.0)	1 (3.6)

**Table 12:** Likelihood of using avatars to participate in avatars in a virtual amputee support group; response to the question, “If you had access to the internet how likely would you be to join a virtual support group using avatars?” (N=54).

Likelihood	All participants, n (%)	Group: 20-39 years, n (%)	Group: 40-59 years, n (%)	Group: 60-82 years, n (%)
Very likely	9 (16.7)	2 (40.0)	5 (23.8)	2 (7.1)
Somewhat likely	5 (9.3)	1 (20.0)	2 (9.5)	2 (7.1)
Neutral	19 (35.2)	1 (20.0)	8 (38.1)	10 (35.7)
Somewhat unlikely	7 (13.0)	0 (0.0)	2 (9.5)	5 (17.9)
Not at all likely	13 (24.1)	1 (20.0)	4 (19.0)	8 (28.6)
I don’t know what teleconferencing is	1 (1.9)	0 (0.0	0 (0.0)	1 (3.6)

## Discussion

### Principal Findings

The purpose of this study was to understand why amputees do or do not participate in support groups and whether there is a role for technology in improving amputee support group engagement. The authors speculated that with the growing prevalence of virtual technology, there was an opportunity for virtual technology to supplement the amputee support group experience. After a failed virtual support group in a previous study [11], it became clear that additional information on support group participation and attitudes toward a “spectrum of increasingly more complex technology” was needed. In the early 1980s, when the use of computer-based training (CBT) was in its infancy, instructors had to take time in a class to provide basic computer literacy—how to turn on and off the computer, save and transfer data, how to use a mouse, etc—skills that are ubiquitous today. We believe that one day, customizing an avatar and navigating a virtual reality environment will also be ubiquitous. Our data show that 100% of amputees in the youngest age group (20-39 years) believe that technology would improve participation in support groups, a finding supported by Taylor et al [13] who used virtual technology with respiratory patients. Although it is important to understand how best to deliver health care, including support to the next generation of health care consumers, we had some unexpected findings.

When examining the text-based participant feedback in Table 4 and the duration of participation in  Figure 1D, we learned that participants had two reasons for joining a support group. The first was to learn skills and improve functionality to regain as much mobility as possible (which includes familiarity with new prostatic technology), and the second was to connect with other people who have had similar experiences. While further research is needed, once the functional goals of a participant are attained, the social aspect seems to become more critical, and if there is no sense of connectivity between participants, amputee support group participation drops over time. The implications of this observation will be examined further in this paper. 

Although the survey covered a lot of ground, there seemed to be four key takeaways regarding amputee participation in support groups:

- The needs of participants in amputee support groups change over time.
- Meeting content needs to be relevant with agendas primarily driven by participants.
- Support group participation is also driven by the desire to
  - Increase functionality by developing skills
  - Become familiar with prosthetic technology
  - Have more than amputation in common with other participants
  - Participate at the designated meeting time and location
- The use of virtual technology should support patients’ needs.

A more detailed discussion of each takeaway is presented below. 

#### The Needs of Participants in Amputee Support Groups Change Over Time

It should come as no surprise that amputee support group participants’ needs change over time. In cases where the initial challenge of living with a prosthesis is met, participants look for deeper connectivity to the other members of the support group. If that does not happen, group participation can be waned.

Just as the Amputee Coalition describes phases of recovery for amputees [14], the data in the study suggest that participation in amputee support groups may follow phases based on time as an amputee and functional capability. Reviewing both the text-based responses in Table 3 and the duration of participation observed in Textbox 1, it may be reasonable to assume that there are phases in support group participation: a short-term phase (≤1 year) with a primary focus on improving functionality and a longer-term phase (>1 year) where connecting with the shared experience of other amputees becomes the primary focus. Table 13 describes how amputee support groups’ goals may vary over time.

From early on, the primary focus of amputees is to learn the life skills needed to return to as normal a life as possible and meet with other amputees who have a shared experience. Over a longer time, as the functional goals are achieved, the primary purpose of continuing participation seems to focus on friendships and relationships with others because of their shared experience, and secondary purpose is to obtain new information on functional issues and prosthetic technology as they become available. If this observation proves to be valid, the implications for support group content and agendas may be significantly impacted, as that agendas should provide content or activities to meet the needs of participants based on where they are in their experience as an amputee.

**Table 13:** Proposed phases of goal priority for amputees in support groups.

Phase	New amputees (≤1 year)	Experienced amputees (>1 year)
Primary goal	Increase functional skills and familiarity with prosthetic technology	Connect with the shared experiences of other amputees
Secondary goal	Connect with the shared experience of other amputees	Enhance existing functional skills and learn about new prosthetic technology

#### Meeting Content Needs to be Relevant With Agendas Primarily Driven by Participants

The decision to participate in an amputee support group is based on the perceived value of what the group has to offer as well as the logistical ability to participate in the meetings. Following the perceived value, or perhaps, as part of that perceived value, the relationships between participants and the meeting content become an important factor for continuing to be engaged in a support group.

For respondents who choose not to participate in a local support group, a common reason for not doing so was feeling disconnected from the content of the meetings. Either the health care professionals drove too much of the agenda or the content was not sufficiently explained, or the reason was relevant to the participants’ needs. While additional research could help gain a better understanding of the opportunity, not surprisingly, it appears that amputees want to be empowered and engaged in their support group meetings by helping to drive the meeting content. This may seem like common sense, but in a number of support group situations, this is not perceived by amputees as common practice.

Additionally, trying to be “all things to all people” with regard to meeting content pits the needs of new amputees against those of seasoned amputees. The data suggest that there may be a need for amputee support groups that provide focus for new amputees as well as a separate focus for “seasoned” amputees. Creating a single agenda for every meeting that serves both groups’ needs may be a significant challenge, but unless there is a large enough population of each category of amputees to support two groups, the meeting agenda may need to have a portion of time dedicated to the needs of the new amputee and a portion of this agenda focused on the more experienced amputee.

These observations also tie into the descriptor we used to label a support group “participant.” Considering that support group participation may occur over time, from starting as a new amputee to becoming an experienced amputee, the label of “patient“ may not apply to a “seasoned“ amputee support group participant. In the article “What should we call the people we work with?” Author John Brinkman observed that early on in the journey of limb loss, individuals are often referred to as “patients” because they may be recovering from an actual illness [15]. The use of the descriptor “patient” also suggests a dependent relationship between amputee and health care providers/prosthetists. However, over time, that relationship changes, where the individual may no longer be “sick,“ so, perhaps, they should no longer be viewed as “patients.”

If the leadership of an amputee support group is largely comprised of health care providers who view its members as “patients” as opposed to “participants,” health care providers may feel justified to be the ones driving the support group agenda and content, that is, they know best what should be covered in a support group meeting. However, once the health care community considers experienced amputees as partners or participants instead of patients, the goals and content of support group meetings can be mutually agreed to. This requires further study but it is possible that participation and engagement in support group meetings could increase with a change in meeting philosophy based on how group participants are defined—as patients or partners.

#### Support Group Participation Driven by Several Factors

Support group participation was driven by the need to improve functionality by developing skills, becoming familiar with prosthetic technology, having more than amputation in common with other amputees, and being available to participate in support group meetings at the designated time and location.

As stated earlier, the research suggests that many people initially join amputee support groups primarily to learn how to live life as an amputee. This can be done through gait clinics and other group activities ranging from swimming to bowling, golf, and many other sport- and hobby-based activities. Once these needs are met, having something in common with the other support group members is important to most amputees’ continued participation in the support group. Although this study only skimmed the surface of elements of commonality, some of the feedback indicated that gender, age, socioeconomic background, and educational background impact the perception of commonality between members of a support group and consequently a connection that may drive continued participation. While support group leaders can easily control meeting content, it is far more challenging to manage the demographic of amputees that participate in support group meetings. Additional research in this area may prove helpful to support group leaders.

Learning about prosthetic technology can be overwhelming for new amputees. Support groups can help when experienced amputees share their experiences with certain technologies. Additionally, product manufacturers and prosthetists might be invited to support group meetings as long as it is understood that the purpose is to objectively inform the audience and not simply promote their products.

In addition, of importance to many amputees was the timing and location challenges of attending amputee support group meetings, which can impact respondents’ level of participation. Trying to find an optimal meeting time and location for all amputees connected to a support group can be very challenging for group leaders. Depending on the size of the group, varying the meeting times or location or the use of communication technology may help with this issue.

#### Virtual Technology Should Support the Participants’ Needs

Respondents indicated a general belief that technology may be used to overcome some of the meeting logistical limitations that were a challenge to some respondents. However, it was also clear that at an individual level, such openness to the use of technology was strongly influenced by the respondents’ comfort level and understanding of specific technology options.

Based on the earlier observations regarding focus on improving amputee functionality, it seems that if communications technology (teleconference, videoconference, or avatar-based virtual world) can assist with improving participant functionality, there is a place for these technologies to supplement support group activities. For example, virtual reality technologies can help amputees by visually demonstrating new skills, safely practice those skills through avatars, gain confidence, and assess functional progress. A limitation of this study was that the technology options included were limited to teleconference, videoconference, or avatar-based virtual world.

In Textbox 1, which presented other reasons for not participating in support groups, participants provided open text responses regarding why they do not participate in amputee support groups. These reasons could be grouped together in several categories such as time, gender bias, perceived value, fear, distance, leadership in the group process, and a sense of commonality. The technology used to create virtual worlds could address a number of these issues.

The issue of time can be addressed by the possibility of offering a variety of meeting times from which support group members could sign up. By not having to travel to participate, the possibility of a larger geography from which to draw participants becomes viable for a virtual group. It may also permit greater flexibility around frequency and timing of meetings.

While no one should have to hide who they are, the anonymous environment afforded by virtual worlds and the wider reach of a support group in a virtual world could allow more members of both genders to participate. According to the Amputee Coalition, male amputees outnumber female amputees by two-thirds [16]. As per the survey results, it is not uncommon for a woman to find herself to be the only female in a face-to-face support group, which may then not focus on her specific gender needs. The potentially wider reach of a virtual environment may allow more women to participate.

In terms of “Perceived Value,” for amputees who feel they do not need support and can make the adjustment on their own or are simply too proud or afraid of showing weakness, the anonymous nature of a virtual support group might open the door to these individuals to encourage them to participate. The same would be true for people who are afraid or embarrassed about their appearance and new situation. The anonymous nature of a virtual environment might make it easier for them to participate.

One of the common themes for not participating in a support group beyond improving one’s functionality with a prosthesis is the social disconnect they feel with other members of the support group. Several respondents said they do not feel they have enough in common with other support group members beyond being amputees. As stated earlier, the use of avatars can provide a level of anonymity that might diminish some of the more obvious differences between participants, at least at the physical level. Being engaged in specific activities like windsurfing or swimming in an online virtual support group setting may provide an environment to help overcome some of those barriers. With the wide range of ages one sees in the survey results, age becomes less significant in an avatar-based environment since the physical limitations that come with age are not restrictive or visible in a virtual world.

Additional research with a stronger focus might provide additional insights regarding the optimal circumstances in which technology, in general, and virtual reality, specifically, may increase amputee support group participation.

### Recommendations to Support Group Leaders

Our recommendations are as follows:

- View your group participants as partners, not just as patients.
- Ask participants what they want to achieve by participating in the group. Do this periodically (not just when a new person joins), since participant goals change over time.
- Engage with support group participants to develop meeting agendas.
- Have meetings to support the development of functional skills for all participants.
- Depending on group size, develop meeting content for both new amputees and experienced amputees:
  - When possible, a portion of each meeting can be used for the needs of new amputees and another portion can be used for the needs of experienced amputees.
  - Alternate meetings where one meeting focuses on the new amputee and the next meeting focuses on experienced amputees can be conducted.
  - Experienced amputees can be used to help and encourage new amputees.
- Look at the support group demographics and brainstorm ways to find common ground between group participants where possible.
- Determine if and how various communication technology options can supplement the support group experience:
  - Make training available to teach participants how to use the technology selected.
  - Develop activities that are engaging for participants using the technology and helps them achieve their goals.
  - Where needed and possible, provide technology support to participants (equipment, financial support, tech support, etc)

While these recommendations will not resolve all issues regarding support group participation, they are based on feedback from a range of support group participants from all walks of life. We believe they can go a long way in enhancing the amputee support group experience as well as improving outcomes for participants.

## Abbreviations

REDCap: Research Electronic Data Capture
